# Feedback that Lands: Exploring How Residents Receive and Judge Feedback During Entrustable Professional Activities

**DOI:** 10.5334/pme.1020

**Published:** 2023-10-20

**Authors:** Natasha Sheikh, Joshua Mehta, Rupal Shah, Ryan Brydges

**Affiliations:** 1 Oakville Trafalgar Memorial Hospital, Oakville, Ontario, Canada; 2 Critical Care Medicine Residency Program, University of Toronto, Toronto, Ontario, Canada; 3 Division of General Internal Medicine, Toronto Western Hospital, University Health Network, Toronto, Ontario, Canada; 4 Faculty Member of the HoPingKong Centre for Excellence in Education and Practice, Toronto Western Hospital, University Health Network, Toronto, Ontario, Canada; 5 Technology-Enabled Education at St. Michael’s Hospital, Unity Health Toronto, CA; 6 Department of Medicine, University of Toronto, Toronto, ON, Canada

## Abstract

**Introduction:**

Receiving feedback from different types of assessors (e.g., senior residents, staff supervisors) may impact trainees’ perceptions of the quantity and quality of data during entrustable professional activity (EPA) assessments. We evaluated the quality of EPA feedback provided by different assessors (senior residents, chief medical residents/subspecialty residents, and staff) and explored residents’ judgements of the value of this feedback.

**Methods:**

From a database of 2228 EPAs, we calculated the frequency of contribution from three assessor groups. We appraised the quality of 60 procedure-related EPAs completed between July 2019 and March 2020 using a modified Completed Clinical Evaluation Report Rating (CCERR) tool. Next, we asked 15 internal medicine residents to sort randomly selected EPAs according to their judgements of value, as an elicitation exercise before a semi-structured interview. Interviews explored participants’ perceptions of quality of written feedback and helpful assessors.

**Results:**

Residents completed over 60% of EPA assessments. We found no difference in modified-CCERR scores between the three groups. When judging EPA feedback value, residents described a process of weighted deliberation, considering perceived assessor characteristics (e.g., credibility, experience with EPA system), actionable written comments, and their own self-assessment.

**Discussion:**

Like other recent studies, we found that residents contributed most to procedure-related EPA assessments. To the established list of factors influencing residents’ judgements of feedback value, we add assessors’ adherence to, and their shared experiences of being assessed within, EPA assessment systems. We focus on the implications for how assessors and leaders can build credibility in themselves and in the practices of EPA assessments.

## Introduction

Competence by Design (CBD) requires supervisors to observe, coach, and assess residents as they perform Entrustable Professional Activities (EPAs) [1]. The intended increase in direct observation and feedback has the potential to foster positive relationships between staff physicians and residents, toward the goal of enhancing residents’ learning and competence [2,3]. Upon implementing CBD in the workplace, however, medical residency programs have reported that significant workload and time constraints often create missed opportunities for direct observation and can delay documentation of EPA assessment data and feedback [4]. In turn, these limitations may undermine residents’ and supervisors’ perceptions of the quality and usefuless of EPA assessments [4,5,6]. In efforts to enhance the timeliness and volume of EPA assessments, senior residents’ have been increasingly asked to assume the supervisor and assessor roles for junior trainees [7,8]. While near-peer feedback may be an under-utilized resource in EPA assessments [9], evidence for its impact is quite mixed. We sought to explore whether and how residents value and interpret the feedback offered by near-peers and staff during their EPA assessments.

Near-peer assessors generate formal data on trainees at similar learning levels [9], which helps the trainees learn, and may also enhance the near-peer’s own learning [10]. Near-peers are often available to provide frequent and accessible feedback, enhancing its timeliness [9]. Some studies indicate that junior trainees find near-peer feedback useful, valuable, and more authentic than staff feedback [11]. Similarities in near-peers’ knowledge levels (i.e., cognitive congruence) and proximity in training (i.e., social congruence) might facilitate trainees comfort in asking questions, acknowledging errors, and accepting feedback [10]. By contrast, other studies suggest that trainees regard near-peer feedback as more lenient compared to feedback from staff, that senior residents want to avoid upsetting junior trainees with low EPA scores or constructive comments, and that such leniency can lead to overinflated EPA scores [5,12]. Hence, including senior residents as near-peer assessors in CBD may lead to variable results in how junior residents receive, value, and act on feedback [5].

Trainees’ judgements of themselves and their assessors, no matter how accurate, tend to impact whether and how they attend to and act on feedback [13,14]. Much research has shown that trainees’ receptivity to and utilization of feedback often depends on their perceived credibility of their assessors [15,16,17,18]. While credibility has been defined variably across social, cultural, and professional contexts, many refer to it as the quality of inspiring belief [16,17,18]. Within medicine, trainees appear to form credibility judgments by identifying and assigning value to their assessor’s characteristics [13,15] including their career stage, their investments in clinical work, their defined role, their strengths as a clinician, and whether they take time to directly observe trainees’ performance [6,13,18,19]. Near-peers may, consequently, be viewed as less credible assessors, if trainees prioritize their assessor’s duration of clinical experience. Another powerful influence on how trainees respond to feedback involves how well the data aligns with their own self-assessments and self-perceptions [20,21]. For instance, one study of medical students found that feedback aligning with their self-assessed performance (i.e., their ‘self-assessment filter’) was judged as most impactful in determining their future learning goals [21]. As near-peers increasingly serve as EPA assessors (completing as much as 61% of EPA assessments) [5,22], we argue that educators and CBD program leaders will benefit from an evaluation of the quantity and quality of EPA assessments from near-peers relative to staff, including how residents receive and value feedback from both sources.

In the present study we aimed to compare near-peer versus staff feedback in the context of procedure-related EPAs performed by junior internal medicine residents. We chose procedures because they allow supervisors to engage in relatively prolonged direct observation, and because we expected assessments of procedures would generate clear, actionable feedback. We aimed to: a) determine the frequency of procedure-related EPAs completed by senior residents compared to staff supervisors, b) explore differences in the quality of procedure-related EPAs completed by staff supervisors compared to senior residents using an established scoring tool, and c) explore how residents’ perceive the impact of assessors’ characteristics (e.g., near-peers or staff, procedural experience) on whether and how they value EPA assessments and related feedback.

## Methods

### Setting

We conducted this study at the University of Toronto core internal medicine (IM) residency program from July 2019 to May 2021. The University transitioned to CBD in July 2019, and within that system residents must perform two formally assessed EPAs per week; senior residents can complete up to 50% of those assessments.

### Study Design

We implemented a three-phase mixed methods design including: (i) descriptive analyses of a database containing procedure-related EPA scores to identify frequency of peer vs. staff assessments, (ii) quality analyses of a subset of documented EPA assessments to compare the feedback content of peers vs. staff, and (iii) an elicitation activity asking residents to sort EPAs by their perceived value, followed by a semi-structured interview to explore how they judge the value of EPA feedback given by near-peers and staff.

### Phase I: Identifying EPA frequency based on assessor type

This phase involved collecting and de-identifying procedure-related EPAs completed between July 2019 and March 2020 from the IM program’s electronic database (2019 Elentra Consortium©). We included EPAs for core procedures that IM residents must become competent in prior to licensure: airway management (bag and mask ventilation), arterial line insertion, arthrocentesis, central line placements, lumbar puncture, paracentesis and thoracentesis. Following de-identification, we sorted and counted the EPAs by assessor type using Excel for Mac (Version 16.16.13): senior residents (post-graduate year (PGY) 2 and 3), chief medical residents (CMR) and subspecialty residents (PGY 4 and above), and staff physicians (Internists and Internal Medicine sub-specialists including clinical associates).

### Phase II: Quality assessment of EPAs

We randomly selected 60 procedure-related EPAs, 20 per assessor group, to undergo quality assessments. To appraise quality, we created a modified version of the established 9-item Completed Clinical Evaluation Report Rating (CCERR) tool (see Appendix 1), initially developed for assessing quality of In-Training Evaluation Reports (ITERs) [23]. Specifically, the tool focuses on factors of quality such as justifying numerical scores, clearly explaining trainee strengths and weaknesses, and providing supportive, concrete recommendations for improvement [23]. Our modifications focused on adapting language to fit the EPA context and led to removal of one question (focused on trainee responsiveness to remediation during a rotation). Two independent raters (NS and RB) then used the 8-item modified-CCERR to rate the quality of the selected EPAs on a 5-point Likert scale. As a source of validity evidence for the modified scale, we calculated the intra-class correlation coefficient to evaluate the inter-rater reliability between the two raters. We averaged the scores from the two raters for each EPA, and then submitted the average score for all 60 EPAs into a one-way analysis of variance (ANOVA), with assessor type as the between subjects factor (SPSS software).

### Phase III: Sorting elicitation exercise and semi-structured interviews

To elicit participants’ lived experiences judging the value of EPA feedback, we presented them with 15 de-identified EPAs of variable quality, with the aim of reproducing the act of receiving EPA forms during their training. Our aim was to prime them to think about how characteristics of assessors and the feedback they provide on EPA forms contribute to their value judgements. We purposively sampled participants from a pool of approximately 75 PGY-1 and PGY-2 IM residents via recruitment emails from the residency program director [9,10]. Residents who volunteered were initially queried on their experiences performing EPAs for procedural skills (to sample diverse perspectives), and subsequently provided informed written consent before participating.

First, we used the mean EPA quality scores generated in Phase II to select five EPA forms each from senior resident, CMR/sub-specialty resident, and staff assessors. For each assessor sub-group, we selected EPA forms that scored approximately 1.0, 0.5, or 0 standard deviations above and below the overall mean score (i.e., to create variation in the quality of feedback). We then wrote a short vignette about each EPA form that included the following information: rotation service, hospital site, clinical setting, type of procedure including its presentation, anatomical focus, case complexity, EPA comments and overall EPA rating (on a 5-point scale).

Second, each participant joined a Zoom call (Zoom Video Communications Inc, San Jose, USA) with either NS or JM guiding data collection. Participants then spent 30-minutes sorting the vignettes into self-defined categories using an online software, Optimal Sort (Optimal Workshop Ltd, Wellington, New Zealand). We asked them to define categories based on their judgements of the credibility and value of the EPA comments.

Third, we conducted a 20- to 60-minute, audio-recorded, one-on-one, semi-structured interview on the same Zoom call. The interview guide (Appendix 2) consisted of open-ended questions asking participants to share how they decided to sort EPAs, as well as their experiences in receiving and judging feedback from different assessors and assessor sub-groups during their own procedure-related EPAs. Interviews were transcribed, anonymized, and reviewed for accuracy before analysis.

### Data analysis

To account for multiple perspectives, four different research team members coded several transcripts separately before the coding guide was developed, refined, and finalized by NS and JM. Our deductive coding was informed by the sensitizing concepts of the factors influencing credibility judgments and self-assessment. We also coded inductively for novel concepts arising in the data. The first author engaged in line-by-line open coding initially. Our team then gathered for multiple meetings to organize, categorize, and refine the coded data into themes based on observed relationships and our collective understanding of the existing evidence.

Throughout data collection and analysis, our team met regularly to discuss developing themes and to make collective decisions on iterative changes to the interview guide and sampling procedure. This process of constant comparison allowed us to explore any unanswered questions or newly identified themes across both new and previously analyzed transcripts [24]. Participant recruitment, data collection, and analysis continued until we judged we had achieved adequate information power, which we defined as the point at which further data did not produce new insights about our identified themes [25]. Transcripts were managed and analyzed using NVivo 12 software (QRS International Pty Ltd, Melbourne, Australia).

### Reflexivity

Our team included researchers with varying clinical, educational and research backgrounds yielding diverse perspectives during data analysis: PGY4 (NS) and PGY3 (JM) residents familiar with completing and receiving EPA assessments, an IM physician (RS) with a Masters in Health Professions Education who completes EPA assessments, and an education scientist (RB) with expertise in mixed methods research methodologies applied to study CBD and feedback practices in health professions education. Team meetings allowed us to challenge each other and to openly discuss how we viewed the data differently and similarly, eventually resulting in a shared understanding, analysis, and interpretation of our data.

### Ethics

Our study protocol was reviewed, approved by, and complied with, the University of Toronto Research Ethics Board.

## Results

### Phase I: EPA frequency by assessor type

Within 8.5-months, 2228 unique procedure-related EPAs were logged in the system. We excluded logs where: the assessor was marked as ‘self’ (N = 397, 17.82%) or as ‘N/A’ (N = 238, 10.68%); the type of procedure was marked ‘other’ (N = 106, 4.76%) or ‘code blue’ (N = 88, 3.95%); and ‘N/A’ was present in both comment sections (N = 433, 19.43%). Of the remaining 966 completed EPAs, the primary assessors included senior residents (N = 257, 26.60%), CMRs/senior subspecialty residents (N = 404, 41.82%) and staff physicians (N = 305, 31.57%).

### Phase II: EPA quality according to assessor type

Our ICC was 0.89 (confidence interval: 0.82 – 0.94), establishing strong inter-rater reliability for the 60 EPA scores. The average modified-CCERR score across all EPAs was 2.87 (1.00, standard deviation) out of 5 points. The average score was 2.75 (0.94) for staff physicians, 2.90 (1.05) for CMR/subspecialty residents, and 2.90 (1.05) for senior residents. Notably, a score of 3.0 indicates ‘acceptable’ quality. Overall, the one-way ANOVA indicated there was no statistically significant difference in these scores according to assessor type (F (2,57) = 0.21, p = 0.81).

### Phase III: Elicitation activity and semi-structured interviews

A total of fifteen residents (six PGY-1 and nine PGY-2) participated in the sorting exercise and interview. Participants tended to sort the vignettes into three categories (9/15), or four categories (4/15), with category labels using terms such as ‘helpful’, ‘useful’, or their antonyms.

**Figure 1:**
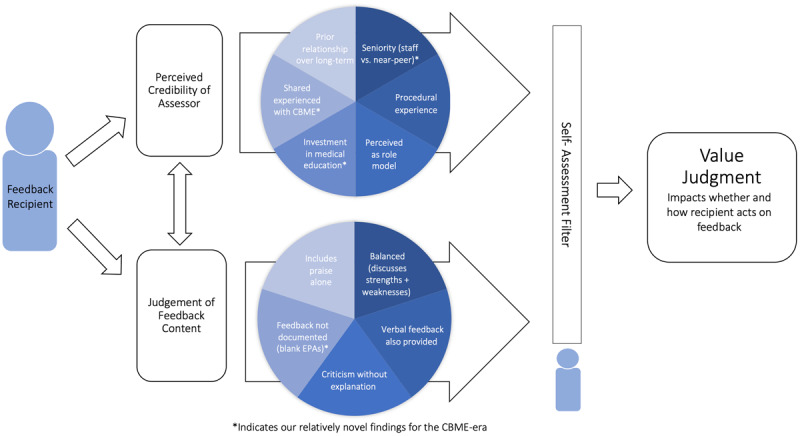
Pictorial represenatation of the deliberative process elicited by residents when receiving and evaluating procedural feedback.

During the interview, participants described some unique factors relating to an assessor’s seniority as a near-peer or staff supervisor as informing their value judgements of EPA feedback. That said, many seemed to focus more on whether individual assessors, regardless of seniority, actively work to relate to learners, and to provide actionable feedback. Participants also described forming their judgements of the feedback they receive from assessors alongside their own self-assessed performance (Figure 1).

### Appraising assessor’s characteristics to assemble perceptions of credibility

#### Where assessor seniority plays a role

Assessors who themselves have functioned as trainees in the CBD system, and thus had also received EPAs, were cited as often providing more substantial feedback:

…when we start as senior residents and fellows, coming into this role of evaluating other people…you feel like you want to do good evaluations…the closer the relationship between the assessor and assessee, the comments tend to be more helpful and, typically, there are more comments. (P9)

In teaching hospitals, the responsibility for conducting bedside procedures often gets shared amongst senior residents, CMRs and subspecialty residents. Subspecialty residents, more senior in training, who have done a procedure more frequently were felt to provide richer feedback:

It’s the fellows who do it a lot more frequently, and so their feedback is very targeted because they can think of very specific scenarios, they’ve been in…[to] troubleshoot that specific issue, and they have the specific solution to it. (P12)

Participants also framed an assessor’s procedural experience according to the accumulation of knowledge over a career, where staff supervisors may be perceived as more credible:

[An] attending would most likely…have seen… a hundred para(centeses)…they’ll have seen ones that haven’t went well…whereas you might get a senior resident who struggled with one but got one, and then their other few were successful where maybe if there was [a complication] would they be in the best position to help me manage that versus my…attending? (P10)

When receiving summative feedback with inherent patient safety implications, participants described judging it as more meaningful when received from staff than from senior residents:

Like probably the most useful, actionable feedback I got… the staff wrote, “well performed, should feel comfortable performing a thoracentesis independently in the future.” And so, to me that was actionable…I felt comfortable doing it and I’ve since gone forward and done them unsupervised because they said I could basically and so that made a big difference. I would be concerned that a senior was just being…reassuring…. so, if I did my first [thoracentesis] supervised by a senior resident or a fellow and they told me to go off on my own… that’s great, but…I need someone more senior than you to sign off on that as a next step. (P1)

We found that participants shared mixed opinions about whether an assessor’s seniority influenced their judgements of the value of procedure-related EPA assessment feedback. Of note, the assessor’s experience being assessed as a trainee in CBD intermingled with their acutely recent, as well as their accumulated, bedside procedural experience.

#### Building relationships with assessors, regardless of seniority

Some residents valued feedback coming from any individual assessor whom they respect:

I have had some feedback from senior residents where they may only be one year ahead of me in training and therefore only have one additional year of experience but based on how I perceive them as a role model and really value the way that they provide clinical care, I will really value the feedback they give to me. (P3)

Participants were also more receptive to constructive feedback from assessors who get to know them in multiple clinical contexts:

…If I know them well… I’m more likely to take their feedback…even more seriously, and I think even not mind so much if they give me constructive and more negative feedback because I know…they’ve seen me in multiple environments and multiple occasions…(P13)

Other participants expressed that having a pre-existing relationship with an assessor increased the likelihood of “getting a good EPA…but not necessarily one that’s providing meaningful feedback” (P5). Another participant noted: “…if there’s a pre-existing relationship, then you may inherently get positive feedback no matter what.” (P10)

Participants cited a higher quality of feedback from assessors who displayed a vested interest in the practice of assessment: “…assessors that are more involved with the EPAs and the curriculum… tend to give the most specific feedback and put the most effort into writing the feedback.” (P7)

Impressions of an assessor’s credibility appeared to be rooted in the depth of shared professional relationships, the assessor’s engagement with education generally and CBD specifically, and the assessor’s procedural experience. When all these facets coalesced into an assessor providing a targeted, rich commentary about an EPA performance, participants’ impressions of credibility rose accordingly. And while an assessor’s seniority matters, participants also seemed willing to allow other relationship-based factors to be weighted more heavily in forming their value judgments of EPA assessment feedback.

### Interplay between feedback content and perceived credibility

Participants described needing to continually reflect on the interplay between their preconceived notions of an assessor’s credibility and their judgement of the quality of feedback content:

I think medicine is a hierarchy and so we all have an inherent bias to be like, “an attending’s feedback may be a bit more helpful than ones from my senior residents.” But…I’ve also gotten feedback from attendings that don’t include feedback, it just includes “successful EPA or not” and I don’t know what I gained from this. (P10**)**

Other participants valued content over pre-conceived notions of credibility: “If someone’s giving me really detailed feedback, it doesn’t matter as much as who it’s from. If it’s really detailed and actionable…it’s probably something to listen to or adhere to”. (P7)

Almost all participants expressed that verbal feedback from any assessor was better than written feedback. It was deemed typically “more honest,” (P8), “more detailed” (P11), and occurred in “real-time” (P1, P11) allowing them to adopt feedback during the procedure. Many participants expressed that the quality of written feedback diminished as time elapsed between task completion and data sharing:

Often, you’re getting feedback during the procedure like, “Oh, try this, do this differently.” And then I think a lot of that is lost as time goes on so especially the longer it takes for somebody to fill out the procedure EPA…the quality of the feedback probably also decreases…(P5)

When judging the credibility of the EPA assessment process, and their assessor’s feedback, participants also appear to factor in the content and timeliness of feedback delivery, alongside their foundational analysis of the assessor’s seniority, identity, and commitments to education.

### Comparing feedback against one’s own perception of performance

When balancing their perceptions of an assessor’s credibility and of feedback content and timeliness, participants also reported focusing on whether feedback aligned with their self-perceptions.

To me I think it’s more about does the feedback make sense more in the context of what I already know… I’ve had the odd staff tell me something that didn’t make any sense to me, it was not what anyone else told me…I just kind of disregarded it. (P1)

Feedback that was incongruent with a resident’s self-assessed performance was regarded as less helpful:

…if you do a procedure that, was totally fine, the patient was totally comfortable, you got fluid, you ultimately achieved what you set out to achieve, and then the person tells you that you’re not entrustable… I would be less likely in general to take that feedback. (P3)

Participants appeared to have a self-assessment filter that they applied to determine the value of each assessor’s feedback:

I think competence to me comes from a mixture of how you feel about your own performance and the feedback you’re getting. I think…if you’ve done enough with senior residents and you feel confident and they were all telling you you’re competent, then that’s probably valid and good. But if a staff does it upfront, you still have to wait and consider your comfort level and…if I had felt uncomfortable doing that thoracentesis, I wouldn’t have taken a staff feedback saying to do it alone and just listened, right? Like…I hope for everyone…if you didn’t feel comfortable doing it…like [myself] [you] wouldn’t have done it independently after that. (P1)

In summary, participants described a process of weighing their perceptions of assessors’ characteristics, of the feedback they provide, and of their own skills and self-assessments, toward judging the credibility and value of EPA assessments. Each assessor’s seniority seemed to have mixed impacts on these perceptions, whereas any assessor committing to building a professional and learning-focused relationship seemed to offer benefit. Indeed, participants described how establishing professional relationships helps them attune their understanding of each assessor’s actual (rather than assumed) expertise, and of each assessor’s investment in educational and assessment praxis; both appearing to enhance participants’ value judgements of the feedback they receive.

## Discussion

In a large pool of procedure-related EPA assessments of junior trainees, we found that senior internal medicine residents assessed about 68% of activities. When assessing the quality of EPA feedback, we used an established tool to determine that comments from senior residents did not differ in quality from those generated by CMR/subspecialty residents, or staff physicians. Our interview data provided mixed perspectives on whether an EPA assessor’s seniority influenced how residents perceived their credibility and value of their feedback. In most cases, participants reported valuing specific, actionable, timely feedback from assessors they have formed relationships with, regardless of assessor seniority. Our findings also affirmed the previously observed interplay between residents’ perceptions of an assessor’s credibility, of the feedback content they receive, and self-perception of their performance. Taken together, this interplay influenced residents’ judgements about the value of procedure-related comments on EPA forms.

In finding that senior residents, CMRs and subspecialty residents assessed most trainees’ performances of procedure-related EPAs, our data reinforce the need to understand whether and how trainees value near-peer feedback. Junior trainees’ increased accessibility to senior residents likely contributed to this finding, which aligns with studies suggesting that accessibility to near-peers facilitates assessment and feedback-seeking processes [26]. Beyond the quantity of EPAs completed by senior residents, our finding that the quality of their narrative feedback did not differ statistically from assessors with greater seniority might yield a mixed interpretation. On one hand, program leaders might be comforted that the observed resident-assessor dominance in quantity is not associated with a cost in quality. Alternatively, these data may cause leaders concern, as they suggest staff supervisors are not offering higher quality feedback, despite their relative experience as educators. In using a modified version of the CCERR tool, the validity evidence we collected suggests the tool can be used for low-stakes appraisals of the quality of EPA comments, like how it has been used to appraise In-Training Evaluation Reports (ITERs) of medical trainees [23]. However, the average CCERR scores, all below 3/5, suggest room for improvement across EPA assessors to provide credible feedback that enhances trainees’ subsequent procedural performance.

Our findings align with prior studies reporting that an assessor’s credibility increases as they: demonstrate clinical expertise [27], invest in directly observing trainees [6,18], invest in building professional (and personal) relationships with trainees [13,23,27], invest in providing timely and constructive feedback [19,28], and acknowledge each trainee’s role [20,23]. An assessor’s seniority certainly appeared to have some (mixed) influence on participants’ perceptions of credibility, though residents’ judgements could be ‘re-balanced’ if assessors made explicit efforts to form relationships and to commit to the EPA assessment process. Firstly, our findings suggest that trainees may be reprioritizing how they selectively establish professional relationships in layered academic settings. Some participants may have gravitated to near-peers as a ready source of quantity and quality in direct observation, and timely and constructive feedback. At the same time, participants noted that staff are gate-keepers in summative assessment decision-making. Researchers have documented the struggle this prioritization of senior residents as assessors can cause, where junior trainees feel conflicted in whether and how they can judge the credibility of formal assessment data (e.g., do high scores reflect my performance or my established positive relationships?) [5]. Secondly, our findings highlight an additional investment that assessors likely must make in the CBD system itself. Senior residents may be emerging as a most trusted assessor group because they likely better understand and have come to accept CBD and EPAs, arguably more so than more senior staff who themselves have not been assessed within this system [5,19,29,30]. That said, the mixed influences that all these factors appear to have on trainees’ perceptions of the credibility and constructiveness of feedback (and of the entire CBD system) remains complicated and complicating for medical educators.

We also found evidence supporting reports that residents interpret the value of feedback by applying a ‘self-assessment filter’. Participants described being more likely to utilize feedback that aligned with their own perception of their performance. Such self-assessments have been notoriously difficult to change [31] as they rely on the trainee’s situated confidence levels, and their perceived experience levels [21]. Hence, we regard this ‘self-assessment filter’ less as something to change and more as an inevitability with which assessors and educators must work. Thus, perhaps one effective way to ‘work with’ self-assessments, however biased, is for assessors to meet the trainee where they are, to become attuned to their needs, to provide timely and constructive feedback, and to invest in becoming a trusted and credible data/feedback source. Research that clarifies these potential mechanisms would be beneficial to CBD program leaders.

Rather than shape our findings into recommendations on who should or should not be assessing EPAs, we aimed to represent the complexity of how feedback is generated, delivered, received, and acted upon in the clinical workplace. Education would help ensure that more CBME program leaders, clinician educators, and residents become aware of the nuanced processes operating through and upon them. Through such training, we believe there would be a rise in the perceived and objective quality of EPA comments, an improved uptake of constructive feedback, and enhancements not only to residents’ clinical skills and knowledge, but also to their relatively poor perceptions of CBME and EPAs.

Study limitations include that our interviewers were themselves residents at the time of data collection, which we chose to mitigate power differentials, yet may have also led to social desirability in participants’ responses. We believe our sorting elicitation exercise likely yielded richer, more situated participant responses. Future studies could explore alternative elicitation techniques, for example, using simulated EPA assessments to account for recall bias. Future studies might also build upon our findings by understanding how residents’ perceived credibility and associated value judgements translate into real world behaviours as they work toward achieving their learning goals.

## Conclusion

To our knowledge, our study has uniquely triangulated quantitative scoring of EPA assessments with resident perceptions of their EPA feedback. The absence of a significant difference in EPA quality scores between the three assessor groups was corroborated by participants’ reporting that their value judgements of EPA feedback did not rely solely on the assessor’s seniority. However, with the shift to CBD and EPA assessments and the resultant increased number of near peers completing assessments, trainees will likely consider each assessor’s seniority as they seek to balance their credibility with the type of feedback they anticipate they will receive. Trainees’ ‘self-assessment filter’, through which they seem to process assessment data and formulate judgements, may be an inevitability that assessors must learn to work with through credibility-building educational practices.

## Additional files

The additional files for this article can be found as follows:

10.5334/pme.1020.s1 Appendix 1. Modified Completed Clinical Evaluation Report Rating (CCERR) Tool to assess EPA quality.

10.5334/pme.1020.s2 Appendix 2. Interview Guide.

## Previous presentations

The International Conference on Residency Education, Montreal, Quebec, Canada, October 29, 2022.

The Canadian Conference of Medical Education, Calgary, Alberta, Canada and Virtual, April 25, 2022.
