# Supplementary material for: Feedback that Lands: Exploring How Residents Receive and Judge Feedback During Entrustable Professional Activities
Source: Perspect Med Educ. 2023 Oct 20;12(1):427–37. doi: 10.5334/pme.1020 (PMC10588547; doi:10.5334/pme.1020)
Supplement: Appendix 1. — Modified Completed Clinical Evaluation Report Rating (CCERR) Tool to assess EPA quality. [file pme-12-1-1020-s1.pdf]

Appendix 1: Modified Completed Clinical Evaluation Report Rating (CCERR) Tool to assess EPA quality

The purpose of this scale is to evaluate the quality of how a clinical evaluation form, such as an Entrustable Professional Activity (EPA) has been *filled out*. With this in mind, please use this scale with regard to *how the form has been completed* rather than the design of the form.

If there is no space for comments on the form, this scale cannot be used. However, if there is a comments section but no comments have been included (i.e. the comments section is blank), please indicate a score of "1" for the questions referring to the comments.

Please rate the following by checking the appropriate box.

	1<br>Not at all	2	3<br>Acceptable	4	5<br>Exemplary
1. Entrustment scale shows sufficient variability to allow identification of relative strengths and weaknesses of the trainee.					
2. Comments are balanced providing both strengths and areas for improvement.					
3. Comments justify the entrustment scale provided.					
4. Clearly explained examples of strengths using specific descriptions (not generalizations) are provided in the comments.					
5. Clearly explained examples of weaknesses using specific descriptions (not generalizations) are provided in the comments.					
6. Concrete recommendations for the trainee to attain a higher level of performance are provided.					
7. Comments are provided in a supportive manner.					

8. Overall, the EPA provides enough detail for an independent reviewer to clearly understand the trainee's performance on the procedure.					

Modified from the original CCERR tool
