# Supplementary material for: Feedback that Lands: Exploring How Residents Receive and Judge Feedback During Entrustable Professional Activities
Source: Perspect Med Educ. 2023 Oct 20;12(1):427–37. doi: 10.5334/pme.1020 (PMC10588547; doi:10.5334/pme.1020)
Supplement: Appendix 2. — Interview Guide. [file pme-12-1-1020-s2.pdf]

## Appendix 2: Interview Guide

### Interview Guide

**Project:** Evaluating the quality and perceived value of procedure-related EPAs completed by senior residents compared to attending physicians within an Internal Medicine program

Date of Interview:

Time of Interview:

Length of Interview:

Site:

Interviewer:

Interviewee:

Interviewee's level of training:

Interviewee's current base hospital:

<u>Instructions for Interviewer:</u>

Before commencing the interview, please explain the purpose/aim of the study, the potential outcomes of the study and any risks to the participant. Reassure the participant that data will be kept strictly confidential and only anonymized data will be available to co-investigators of the study. Advise the interviewee that the duration of the interview will be approximately 45-60 minutes and that the interview will be recorded/transcribed. Review the consent form with them and note the date/time that verbal consent was obtained if they agree to participate in the study. Inform participants that they may decline to answer any questions or stop the interview at any time.

***General questions:***

1. *What is your current year in the internal medicine program? What is your base site and how many rotations have you completed thus far?*
2. *Have you received procedure-related EPAs to date in residency? If so, how many do you think you've completed to date (a rough estimate)?*

***Post-exercise questions:***

1. *Based on the exercise you just completed, please describe how you decided to sort the EPA assessments into the provided categories?*
2. *When reading through the EPA feedback, what did you use to form your judgements about whether they were helpful or not?*
3. *Would knowing the assessor's role - staff vs. senior resident vs. CMR/subspecialty resident - impact how you sorted the EPAs? Please elaborate.*

***Authentic procedure-related EPA-related questions:***

1. *What feedback associated with procedure-related EPAs have you found most helpful? Least helpful? Why is that?*
2. *Have you noticed any differences between the verbal feedback provided in the moment and the feedback provided in the comments section? If so, can you elaborate?*
    a. *Probe: Have you seen this with specific assessor groups?*
3. *Has the amount of elapsed time between the procedure and completion of the EPA influenced the written feedback you received in the EPA? Please elaborate.*
    a. *Probe: Has this differed at all between assessor groups?*

4. *How have the circumstances surrounding a procedure influenced the EPA feedback you received?*
    a. *Probe: Has this differed at all between assessor groups?*
5. *How does your relationship with the assessor impact your experience with procedure-related EPAs?*
    a. *Probe: How does it impact how you receive the feedback? Do you interpret the feedback differently in any way?*
6. *Based on all the procedure-related EPAs you have completed thus far in your residency training, which assessor groups have completed them? Why do you think that is?*
    a. *How would you describe your experiences with having procedure-related EPAs completed by each assessor group? Has the assessor's role impacted the feedback you receive in any way? How so?*
    b. *Does the assessor's role impact how you interpret and use the feedback you receive? Why or why not?*
7. *Have you found certain assessor groups to be more willing to complete EPAs for you? If so, which assessor group(s) are these? Please elaborate.*
8. *Have your perceptions on the value of feedback from different assessor groups, such as staff versus senior residents, changed over time? Why or why not?*
9. *Is there anything else you'd like to share about procedure-related EPAs?*
